# Hepatitis C Virus Infection in the PERSIAN Guilan Cohort Study Population: Intrafamilial Transmission Incidence and Response to SOVODAK‐Based Therapy

**DOI:** 10.1002/hsr2.72619

**Published:** 2026-06-09

**Authors:** Farahnaz Joukar, Sara Yeganeh, Narges Eslami, Fariborz Mansour‐Ghanaei

**Affiliations:** ^1^ Gastrointestinal and Liver Diseases Research Center, Razi Hospital Guilan University of Medical Sciences Rasht Iran

**Keywords:** HCV, hepatitis C virus, intrafamilial transmission, sofosbuvir–daclatasvir, SOVODAK

## Abstract

**Background:**

Hepatitis C virus (HCV) infection is one of the leading causes of liver‐related diseases such as cirrhosis and hepatocellular carcinoma. Despite ongoing efforts, no effective vaccine has been developed to date due to the virus's high mutation rate and extensive genetic variability. This study aimed to determine the prevalence of HCV infection among family members of HCV‐positive index cases, within related risk factors in them. Additionally, the study evaluated the therapeutic efficacy of the combination drug Sovodak (sofosbuvir–daclatasvir) based on HCV genotype.

**Methods:**

This study is a descriptive cross‐sectional study conducted on the families of individuals with Hepatitis C (12 people) within the Guilan cohort population. Twenty family members of HCV‐positive individuals were enrolled. Data were collected using structured questionnaires that included socio‐demographic and clinical information. Blood samples were drawn from each participant, and sera were separated for serological analysis. HCV antibody‐positive samples were assessed by HCV‐RNA detection. Subsequently, positive samples underwent HCV genotyping. Then, HCV‐positive individuals were referred in Phase 3 clinical trial with ID: NCT03200184. for treatment and follow‐up. Treatment consisted of Sovodak (a combination of sofosbuvir 400 mg and daclatasvir 60 mg). The clinical and diagnostic effectiveness of the treatment was evaluated 12 weeks after therapy initiation.

**Results:**

Out of the total cases, two were HCV antibody positive, and one of them was a 13‐year‐old girl who tested positive for HCV RNA by PCR. Her genotype was 1a, which matched her mother's genotype. This patient was successfully treated with Sovodak. The post‐treatment HCV RNA results were negative, indicating a sustained virologic response (SVR = 12).

**Conclusion:**

Evidence of intrafamilial transmission was observed in this study, its frequency was extremely low and statistically negligible. Shared household items, although seemingly unlikely, such as toothpaste, may serve as a potential route of transmission within families, independent of direct interpersonal contact.

**Abbreviations**
ALP: alkaline phosphatase
ALT: alanine aminotransferase
AST: aspartate aminotransferase
CBC: complete blood count
DAAs: direct‐acting antivirals
ECLIA: electrochemiluminescence immunoassay
HCC: hepatocellular carcinoma
HCV: hepatitis C virus
LoD: limit of detection
real‐time RT‐PCR: real‐time reverse transcription polymerase chain reaction
SD: standard deviation
SOVODAK: sofosbuvir 400 mg and daclatasvir 60 mg
SVR: sustained virological response
WHO: World Health Organization

## 1 Introduction

Hepatitis C virus (HCV) is a single‐stranded RNA virus belonging to the Flaviviridae family and is primarily transmitted through exposure to infected blood. It remains one of the leading causes of chronic liver disease worldwide, with an estimated 3% of the global population chronically infected [1]. Over the past three decades, HCV has emerged as a significant global health challenge, currently ranking as the seventh leading cause of mortality worldwide [2]. According to the World Health Organization [3], approximately 71 million people worldwide are living with hepatitis C virus (HCV) infection. Each year, an estimated 399,000 deaths are attributed to complications of the disease, primarily cirrhosis and hepatocellular carcinoma [3].

A large proportion of individuals living with HCV reside in low and middle‐income countries [4]. In Iran, viral hepatitis, particularly HCV, poses a substantial public health burden. A national burden‐of‐disease study reported a declining trend in HCV prevalence between 2010 and 2016, with the prevalence in the general population remaining below 1%, reflecting the effectiveness of national control efforts [5]. However, regional variations in prevalence have been observed. For instance, Kermanshah Province has reported a prevalence rate of approximately 0.85% [6, 7].

Genomic analysis of HCV, including sequencing of the 5′ untranslated region (5′ UTR) and non‐structural protein‐coding regions, has enabled the classification of HCV into at least 11 genotypes, each differing by approximately 30%–50% at the nucleotide level [8]. Due to HCV's high mutation rate, each genotype comprises numerous subtypes (currently at least 93) differing in their geographic distribution, disease progression, and response to therapy [6, 8]. Globally, genotypes 1, 2, and 3 are the most widespread, with genotype 3 being particularly prevalent in South Asia [2, 6]. Common subtypes worldwide include 1a, 1b, 2a, and 3a. In Iran, genotype 3a is the most prevalent, followed by subtype 1a [2, 6].

The high mutation rate of HCV remains a major barrier to the development of an effective vaccine. There is currently no licensed vaccine against HCV, and immunoglobulin therapy following exposure is not an effective prophylactic strategy [9]. Given the substantial geographic variation in genotype distribution and the differences in treatment response, genotyping is essential before initiating antiviral therapy [10]. Drug resistance patterns and the efficacy of direct‐acting antivirals (DAAs) vary across genotypes, underscoring the importance of genotype‐guided personalized treatment strategies [11, 12].

HCV is transmitted primarily through bloodborne routes, including transfusion of infected blood products, surgical procedures, injection drug use, sexual contact, and tattooing [13]. Sexual transmission is relatively uncommon, and the precise mechanisms are not fully understood; nonetheless, risk increases in individuals with multiple sexual partners [14, 15]. Breastfeeding has not been shown to elevate the risk of vertical transmission from mother to infant. Elevated risk groups include patients undergoing dialysis, those with hemophilia or thalassemia, organ transplant recipients, and cancer patients receiving blood transfusions during chemotherapy. Healthcare workers also face occupational exposure risk [5]. The virus is not spread via breastfeeding, sneezing, hugging, coughing, consumption of food or water, sharing eating utensils, or casual contact [9]. Beyond bloodborne transmission and established risk factors, additional transmission routes may exist, particularly in certain patient populations. Epidemiological research has documented familial clusters of HCV, raising questions about the possibility of horizontal transmission to spouses or other household members in contact with HCV‐positive individuals; however, this remains a matter of ongoing investigation [16, 17].

Iran, a multi‐ethnic country located in the Middle East, a region with a relatively high prevalence of HCV infection, lacks a nationwide, population‐based surveillance system, making it challenging to accurately estimate the disease burden and the potential for horizontal transmission. Considering the varied transmission pathways and the differences in HCV prevalence across regions, this study aimed to assess the prevalence of HCV infection associated with household contact and to identify relevant risk factors. Specifically, the study examined, for the first time, the occurrence of hepatitis C among household members and regular contacts of HCV‐infected individuals within the PERSIAN Guilan Cohort population. This cohort represents one of 18 distinct study regions within a large, population‐based prospective cohort conducted in northern Iran. Subsequently, the patients received treatment, and follow‐up assessments were carried out to monitor outcomes

## 2 Study Design and Methods

### 2.1 Study Participations

This study is an analytical cross‐sectional study conducted on the families of individuals with Hepatitis C (12 people) within the PERSIAN Guilan cohort population (from October 8, 2014, to January 20, 2017, in Some'e Sara County, involving both men and women aged 35–70 years). The primary data related to the Guilan cohort profile has been published in detail [18]. Twelve out of 10,520 individuals were identified as HCV‐positive [19]. Among these, 10 participants expressed willingness to undergo treatment with Sovodak and participate in follow‐up assessments, based on the study by Merat et al. (a multicenter Phase 3 clinical trial; ClinicalTrials.gov ID: NCT03200184) [20].

A total of 20 family members of the HCV‐positive individuals, each of whom had been living in the same household and in regular contact with the index case for at least 1 year, were enrolled in the study after obtaining informed consent. These family members included first‐degree relatives such as parents, siblings, spouses, and children. All underwent diagnostic evaluation and treatment procedures. The protocol of this study was reviewed and approved by the Ethics Committee of Guilan University of Medical Sciences (Ethics code: IR.GUMS.REC.1397.164). All procedures performed in this study were conducted in accordance with the ethical standards of the institutional and national research committee, as well as the principles outlined in the Declaration of Helsinki.

Inclusion criteria encompassed: All family members of HCV‐positive individuals, aged 12–75 years, with no prior history of antiviral treatment, and willingness to participate as documented by informed consent.

### 2.2 Data Collection

Data collection was performed using a structured questionnaire aimed at identifying potential intra‐familial transmission risk factors and probable modes of HCV transmission. The questionnaire covered: Demographic variables: age, sex, place of residence, and occupation; medical and behavioral risk factors, including: history of injecting drug use, blood transfusion or receipt of blood products, surgery with transfusion, tattooing, hemodialysis, cupping (hijama), needle‐stick injuries, high‐risk sexual behaviors, endoscopy, colonoscopy, incarceration, dental procedures, employment in high‐risk occupations, such as: physicians, dentists, nurses, healthcare workers, dialysis center personnel, laboratory technicians, barbers, long‐haul transit drivers. All collected data were used to evaluate the patterns and risk factors associated with intra‐familial transmission of HCV and to inform targeted public health interventions.

### 2.3 Sampling, Immunological, and Virological Assessments

Blood samples (5–10 mL) were collected from participants and promptly sent to the laboratory. Serum was separated from the whole blood samples and analyzed for the presence of anti‐HCV antibodies among family members of HCV‐infected patients using the Cobas anti‐HCV assay, based on electrochemiluminescence immunoassay (ECLIA) technology, with the Cobas e 411 analyzer (Roche Diagnostics, Germany). Samples that tested positive for anti‐HCV antibodies underwent confirmatory HCV RNA PCR testing. HCV RNA was extracted from serum samples obtained from HCV‐positive patients using a commercial viral RNA extraction kit (Roche, Germany), following the manufacturer's protocol. Quantification of HCV RNA copies was performed by real‐time reverse transcription polymerase chain reaction (real‐time RT‐PCR) utilizing the COBAS AmpliPrep/COBAS TaqMan HCV Test (Roche Diagnostics, Germany).

This assay has a limit of detection (LoD) equivalent to the lower limit of quantification (LLoQ) at 25 IU/mL, as specified by the manufacturer. For samples with confirmed HCV RNA positivity, genotyping was performed using HCV‐specific primers targeting the conserved core region of the virus.

### 2.4 Therapeutic Procedure and Follow‐up

All participants who tested positive for HCV RNA were treated with Sovodak (Rojan, Pharma, Tehran, Iran), a fixed‐dose oral tablet combination of sofosbuvir 400 mg and daclatasvir 60 mg, administered once daily for 12 weeks based on Merat et al.'s study with ClinicalTrials.gov ID: NCT03200184 [20]. For patients diagnosed with liver cirrhosis based on FibroScan results, ribavirin was added to the treatment regimen at a dosage of 1000 mg per day for individuals weighing less than 75 kg, and 1200 mg per day for those weighing 75 kg or more, administered in 5–6 divided doses of 200 mg each [20]. Patients were monitored at regular intervals for adverse events and medication adherence, both in‐person and via telephone consultations.

During the treatment period, participants attended monthly check‐ups, with a final evaluation conducted 3 months after the therapy concluded. For those receiving ribavirin, an additional visit was scheduled at the 2‐week mark to monitor for signs of anemia. At each visit, patients were asked about any side effects and whether they were following the prescribed treatment regimen. Blood tests were performed regularly, including complete blood counts and assessments of liver enzymes (ALT and AST), bilirubin (both total and direct), and creatinine levels. Treatment was halted if ALT rose to 10 times above baseline levels or if creatinine levels increased to the point where the estimated glomerular filtration rate (eGFR) dropped below 30 mL/min/1.73 m^2^. In the ribavirin group, a hemoglobin reduction of 2 g/dL or more led to discontinuation.

The primary outcome evaluated was the sustained virologic response at 12 weeks after treatment completion (SVR12), defined as the absence of detectable hepatitis C virus RNA using a highly sensitive PCR test with a detection threshold of less than 25 IU/mL. Adverse events were classified into three levels: mild (self‐limiting or not requiring medical attention), moderate (needing medical treatment but not affecting therapy continuation), and severe (leading to the discontinuation of hepatitis C treatment).

**Figure 1:**
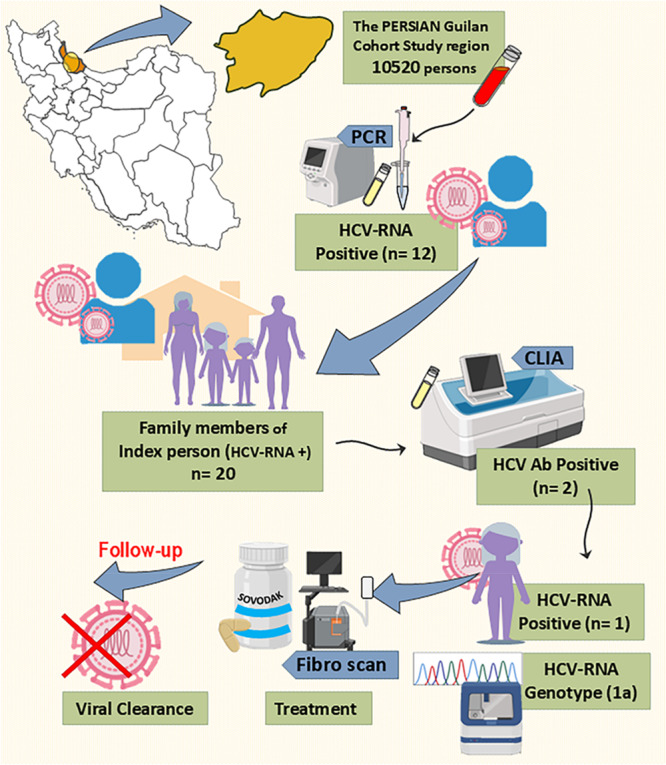
Schematic diagram illustrating the study process, from participant sample collection to treatment outcome assessment.

Figure 1 summarizes the process of conducting this study.

### 2.5 Informed Consent

After informing each participant of the study's purpose and importance, written informed consent was obtained from all participants (patients and family members) prior to their enrollment in the study. To ensure participant confidentiality, alphanumeric codes based on participant names were used, and no personally identifiable information was recorded on the questionnaires.

### 2.6 Statistical Analysis

Statistical analyses were performed using SPSS Statistics software, version 22 (SPSS Inc., Chicago, IL, USA). Descriptive statistics for numerical variables are presented as mean ± standard deviation (SD). Categorical variables are expressed as absolute frequencies and percentages.

## 3 Results

### 3.1 Demographic and Clinical Characteristics of HCV Infected Persons and Intrafamilial Transmission Route

Based on serological testing for total anti‐HCV antibodies (indicating exposure to HCV), among 20 relatives of HCV‐positive patients, only two individuals tested positive for anti‐HCV antibodies. Comparative analysis of risk factors among all family members of the index case (HCV RNA‐positive) suggested that female gender, history of dental procedures, and lower awareness due to limited educational attainment could be considered potential risk factors of significance.

Among these two anti‐HCV‐positive individuals, qPCR confirmed active HCV RNA infection in only one case. This was observed in a 13‐year‐old girl whose viral genotype (1a) matched that of her mother. The clinical characteristics and risk factors associated with horizontal transmission in these patients are summarized in Table 1. Importantly, the most notable finding of this study was the pattern of intrafamilial transmission from mother to daughter. The daughter not only tested positive for HCV RNA with a significant viral load but also exhibited close genotypic similarity to her mother's strain. Among the identified and evaluated risk factors, a history of dental interventions and the shared use of toothpaste were particularly noteworthy.

**Table 1:** Clinical characteristics of a patient with an intrafamilial transmission route.

Parameters	HCV Ab+ (*n* = 2)	
Age (years)	13	43
Sex (male/female)	Female	Female
Place of residence	Urban	Rural
Occupation	Student	Housewife
Marital status	Single	Widow
History of injecting drug use	(−)	(−)
Blood transfusion	(−)	(−)
receipt of blood products	(−)	(−)
Surgery with transfusion	(−)	(+)
Tattooing	(−)	(+)
Hemodialysis	(−)	(−)
Cupping (Hijama)	(−)	(−)
Needle‐stick injuries	(−)	(−)
High‐risk sexual behaviors	(−)	(‐)
Endoscopy/colonoscopy	(−)	(+)
Incarceration	(−)	(−)
Dental procedures	(+)	(+)
Employment in high‐risk occupations	(−)	(−)
First‐degree relative of the HCV‐positive individual	Daughter of a mother with HCV+	Sister of brother with HCV+
Shared personal items with the HCV‐positive individual/type	(+), toothpaste	(−)
Viral load of HCV (IU/mL)	838,980	0
HCV genotype	1a	0

The therapeutic outcome of treatment with Sovodak was highly satisfactory, with minimal adverse effects reported. By Week 12 of therapy, the patient's HCV‐RNA became undetectable, demonstrating a virologic response. Furthermore, the SVR, defined as the complete clearance of the virus from the body, was confirmed at both Week 12 and Week 24 post‐treatment through molecular assays and FibroScan evaluation, thereby verifying the long‐term efficacy of the regimen.

## 4 Discussion

The importance of considering HCV as a blood‐borne infection lies in the necessity of strict adherence to safety protocols. This becomes particularly relevant given both the virus's high environmental stability remaining viable for more than 6 weeks [21] and its potential for horizontal transmission through contact with secretions from infected individuals, especially among family members [22]. Household contacts, including spouses, children, parents, and siblings, share living spaces regardless of genetic background, thereby increasing the risk of transmission [23].

Interfamilial transmission has been recognized as a major risk factor in developing countries and in regions with high HCV prevalence. However, it remains under investigation due to methodological and sociocultural challenges, such as limited access to early‐stage infection samples, difficulties in determining the approximate time of exposure, and variations in study design across different populations [4, 23]. Although tracking HCV transmission within households is complicated by the presence of multiple potential routes of infection, numerous studies have emphasized that familial clustering contributes significantly to the overall burden of HCV [24, 25].

In the present study, conducted within the PERSIAN Guilan Cohort Study population of Guilan Province, northern Iran, the seroprevalence of HCV infection among family members of index cases was 10% (2 of 20 individuals). Reported estimates of intrafamilial transmission rates in Iran vary considerably, ranging from 1.3% in western and southern regions (Hamadan and Khuzestan Provinces) to as high as 32% in southern Iran (Fars Province) [24, 26, 27]. These findings are consistent with Iran's classification as a country with intermediate HCV prevalence, with an overall prevalence of 1.26% in the general population and 0.11% within the Gilan Persian Cohort [19, 28]. Nonetheless, the relatively small sample size in our study should be considered when interpreting these results.

Comparable intrafamilial transmission rates have been reported globally: 30.1% in Pakistan [29], 20% in Egypt [30], 9% in Italy [31], and 7.7% in Yemen [25]. This variability may be attributed to sociocultural practices, behavioral differences, regional prevalence rates, and disparities in diagnostic methodologies (serological vs. molecular assays) [32, 33]. In our study, genotype 1a was identified as the transmitted strain in a documented mother‐to‐child case. This aligns with previous reports highlighting genotype 1a as the predominant circulating strain in Iran [2, 28], frequently associated with intrafamilial spread [34], followed by genotype 3a [2]. However, genotype distribution may evolve over time depending on transmission routes [34].

All HCV‐seropositive family members of the index case in our study were female, consistent with other reports indicating higher infection rates among female relatives [35, 36]. Nevertheless, some studies from Iran and Yemen have shown higher rates in males [24, 25], often attributed to behavioral and occupational risk factors such as shared razors, toothbrushes, or tattooing practices [37]. Age also appears to influence susceptibility: while most studies report higher infection rates among relatives older than 30 years [25, 35, 38], some cases have been documented in individuals ≤ 19 years [39, 40]. In our study, seropositive family members of the index case were aged 43 and 13 years (with active infection). Such discrepancies may reflect differences in household exposure patterns and the intensity of contact with the index case rather than inherent biological susceptibility.

The relationship type (e.g., mother–daughter, brother–sister) did not appear to influence transmission risk in this study. However, other investigations have reported variable rates of infection among different kinship groups [35, 39], with higher clustering frequently observed among brothers [24, 25]. Close daily interactions and shared use of personal items, especially grooming tools, may account for these patterns [37]. Some studies have also noted increased risk of infection when the index case is female [25, 41], though such findings may be influenced by sample size and the gender of index cases.

Dental procedures emerged as another potential risk factor for intrafamilial HCV transmission in our study. Previous research has also highlighted dental clinics as a significant source of exposure, given the unavoidable risk of blood contact during dental interventions [42, 43]. Unfortunately, many patients fail to disclose their HCV status, and dental practitioners rarely inquire about infection history, thereby heightening risk. Nonetheless, the contribution of dental visits to household transmission is most plausible when family members attend the same dental clinic and undergo repeated procedures.

Educational level was also associated with infection status. Infected family members had education levels below high school, consistent with findings from Yemen and Iran [24, 25]. Lower education may limit awareness of personal hygiene and disease prevention, while also correlating with closer household contact. For example, one infected individual in our study reported sharing toothpaste with the index case, likely reflecting a lack of awareness regarding HCV's environmental persistence (over 6 weeks) [21]. Previous studies have confirmed the risk associated with sharing towels, razors, needles, or toothbrushes [24, 44]. To our knowledge, this is the first report implicating shared toothpaste as a potential risk factor for intrafamilial HCV transmission.

The therapeutic outcome of treatment with Sovodak for HCV genotype 1a was highly satisfactory, with only minimal adverse events reported. The clinical efficacy of this combination regimen has also been well‐documented in previous studies [45].

Among the three WHO‐recommended first‐line DAA regimens with pan‐genotypic coverage, Sofosbuvir–Daclatasvir (SOVODAK), is the preferred option in many low‐ and middle‐income countries, including Iran. This fixed‐dose combination (sofosbuvir 400 mg + daclatasvir 60 mg) is effective against all major genotypes [46, 47].

Sofosbuvir is a nucleotide analogue inhibitor of the HCV NS5B RNA‐dependent RNA polymerase, while daclatasvir is a potent NS5A inhibitor with pan‐genotypic activity [12, 20].

**Figure 2:**
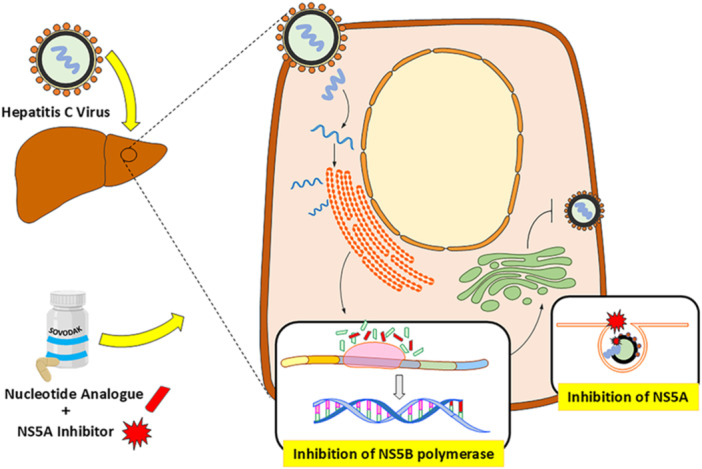
Molecular mechanism underlying Sovodak therapy, which inhibits viral replication and assembly. (Sovodak), a fixed‐dose combination therapy consisting of sofosbuvir and daclatasvir, targets two essential viral proteins of the hepatitis C virus (HCV). Sofosbuvir, a novel nucleotide analogue inhibitor of the NS5B RNA‐dependent RNA polymerase, interferes with viral RNA replication by inducing premature chain termination. Daclatasvir, an NS5A inhibitor, binds to domain I of the NS5A protein, thereby disrupting the assembly of the replication complex and inhibiting both RNA synthesis and virion assembly. The complementary mechanisms of action of these agents result in a highly effective antiviral regimen with a broad genotypic coverage and a reduced likelihood of resistance development.

Figure 2 shows the molecular mechanism underlying Sovodak therapy, which inhibits viral replication.

This study has several limitations, including the small sample size, uncertainty regarding the duration of infection in index cases, incomplete information about the timing of household exposure, and lack of data on co‐infections with HIV–HCV or HBV–HCV, which are known to influence HCV transmission.

In conclusion, our findings underscore the importance of screening family members, partners, and close contacts of HCV index cases. Such measures facilitate early detection and linkage to care. Additionally, counseling newly diagnosed patients regardless of education level may enhance awareness of transmission routes and mitigate HCV‐associated stigma, which often deters disclosure to family members and community networks. Addressing stigma is essential to encourage open communication, reduce social isolation, and strengthen preventive practices.

## 5 Conclusion

Although the prevalence of hepatitis C virus (HCV) infection among family members in the PERSIAN Guilan Cohort Study population, northern Iran, was lower compared to most global studies, it should not be considered negligible. In this study, the main risk factors associated with intrafamilial transmission were identified as sharing toothpaste with an infected individual, Dental procedures, and insufficient awareness, particularly in individuals with lower educational attainment. Sofosbuvir‐Daclatasvir (SOVODAK) demonstrated effective antiviral activity against HCV genotype 1a, supporting its use by clinicians as a viable therapeutic option for viral clearance. Furthermore, the cultural stigma surrounding HCV within families, which often leads the index case to conceal their diagnosis, should not be underestimated as a contributing factor in disease management and transmission dynamics.

## Author Contributions

**Farahnaz Joukar:** conceptualization, investigation, writing – review and editing, methodology, validation, data curation, software, and formal analysis. **Sara Yeganeh:** investigation, writing – original draft, writing – review and editing, validation, methodology, data curation, and software. **Narges Eslami:** investigation, writing – original draft, writing – review and editing, validation, data curation, methodology, and visualization. **Fariborz Mansour‐Ghanaei:** conceptualization, writing – review and editing, supervision, data curation, project administration, methodology, validation, and investigation.

## Funding

The authors have nothing to report.

## Conflicts of Interest

The authors declare no conflicts of interest.

## Data Availability

The data that support the findings of this study are available from the corresponding author upon reasonable request. The data sets used and/or analyzed during the present study are available from the corresponding author upon reasonable request.
